# Correction: The mediating role of psychological resilience on the negative effect of pain in patients with rheumatoid arthritis: a cross-sectional study

**DOI:** 10.1371/journal.pone.0318945

**Published:** 2025-02-03

**Authors:** The PLOS One Staff

## Notice of Republication

This article was republished on January 24, 2025, to correct an error in the title that was introduced during the typesetting process. The publisher apologizes for the error. Please download this article again to view the correct version. The originally published, uncorrected article and the republished, corrected articles are provided here for reference.

## Supporting information

S1 File Originally published, uncorrected article. (PDF)

S2 File Republished, corrected article. (PDF)
