# Supplementary material for: Correction: The mediating role of psychological resilience on the negative effect of pain in patients with rheumatoid arthritis: a cross-sectional study
Source: PLoS One. 2025 Feb 3;20(2):e0318945. doi: 10.1371/journal.pone.0318945 (PMC11790146; doi:10.1371/journal.pone.0318945)
Supplement: S2 File — (PDF) [file pone.0318945.s002.pdf]

PLOS ONE

RESEARCH ARTICLE

The mediating role of psychological resilience on the negative effect of pain in patients with rheumatoid arthritis: A cross-sectional study

Shuang Xu[1☯]*, Qiongyu Zhang[2☯], Jiayan Zhou[2☯]

1 Department of Psychology, College of Humanities and Management, Guizhou University of Traditional Chinese Medicine, Guiyang, Guizhou, China, **2** Department of Rheumatology and Immunology, Second Affiliated Hospital of Guizhou University of Traditional Chinese Medicine, Guiyang, China

☯ These authors contributed equally to this work.
* xsgzutcm1999@163.com

Check for updates

OPEN ACCESS

Citation: Xu S, Zhang Q, Zhou J (2023) The mediating role of psychological resilience on the negative effect of pain in patients with rheumatoid arthritis: A cross-sectional study. PLoS ONE 18(12): e0295255. https://doi.org/10.1371/journal.pone.0295255

Editor: Giuseppe Marano, Fondazione Policlinico Universitario Agostino Gemelli IRCCS, Universita' Cattolica del Sacro Cuore, ITALY

Received: April 15, 2023

Accepted: November 18, 2023

Published: December 1, 2023

Copyright: © 2023 Xu et al. This is an open access article distributed under the terms of the Creative Commons Attribution License, which permits unrestricted use, distribution, and reproduction in any medium, provided the original author and source are credited.

Data Availability Statement: All relevant data are within the paper and its Supporting Information files.

Funding: The authors received no specific funding for this work.

Competing interests: The authors have declared that no competing interests exist.

Abstract

The objective of this study was to investigate the direct effects of pain-induced depression and anxiety, as well as the mediating role of psychological resilience, on the psychological distress associated with rheumatoid arthritis. The method involved a sample of 196 patients with rheumatoid arthritis and applied the Hospital Anxiety and Depression Scale, Connor–Davidson Resilience Scale, and visual analog scale for pain. Bivariate and path analyses were performed, and a multiple mediational model was utilized. Results showed that all correlations among study variables were significant ($p < 0.01$). A partial mediation effect of psychological resilience was observed, and direct effects among the variables (pain, psychological resilience, anxiety, and depression) were statistically significant, including the direct effect of psychological resilience on depression and anxiety. The indirect effects of pain through psychological resilience on depression and anxiety were also significant. Thus, the results suggest that psychological resilience partially mediates the effects of pain-induced anxiety and depression in patients with rheumatoid arthritis.

Introduction

Persistent and disabling pain is the hallmark of rheumatoid arthritis (RA) [1], a chronic inflammatory disease that mainly affects the joints. According to Covic et al. [2], the estimated prevalence of RA is 0.5%–1%, and it affects between 0.2% and 0.93% of adults in China [3]. The clinical symptoms of RA include joint pain and swelling, early morning stiffness, and fatigue [4]. It has been well-documented that recurrent pain is a key barrier to physical functioning for patients with RA [5]. In addition, the relationship between pain, depression, and anxiety has been widely demonstrated [6–9], and patients with RA are at greater risk of developing mood disorders due to the chronic course of the disease [10]. Patients with RA are frequently affected by depression and anxiety comorbidities, with depression prevalence rates ranging from 16.8% to 38.8%, based on clinical assessments [11]. A significant prevalence of anxiety in RA, ranging from 21% to 70%, has also been reported [12].

Despite this, some patients with RA manage to live with pain, maintaining their psychological well-being and physical health [13]. This optimistic response to adversity is an example of *psychological resilience* (hereinafter "resilience"), the ability to recover from disease-related stressors and maintain optimal functioning. Resilience is reported to protect people against the negative effects of chronic disease [14–16]. Additionally, the protective effects of resilience against illness-related stressors, severe symptoms, and mental disorders have been widely reported [16,17].

As a new paradigm, resilience has been proposed to promote successful adaptation to chronic pain [17,18]. Given these findings, it seems possible that some patients, although they suffer from RA, adapt well to living with the disease, especially coping with its related symptoms. Several studies have demonstrated the mediating role of resilience on the negative effect of pain-induced psychological distress, such as depression and anxiety [10,19,20]. However, in patients with RA, resilience has not been examined simultaneously for its potential role in mediating pain-induced depression and anxiety.

The current study used a cross-sectional approach. Considering that anxiety and depression are common comorbidities of RA, mainly due to arthritic pain [4], we hypothesized that resilience mediates the effects of pain-induced depression and anxiety. Specifically, 1) pain has a significant direct effect on depression and anxiety, and 2) pain's indirect effect on depression and anxiety is mediated by resilience. These hypotheses were tested using a multiple mediation model, highlighting variables' interconnections. Investigating the mediating role of resilience can offer more approaches to RA treatment, enhance patients' psychological well-being, and assist them in better managing their pain and health.

Materials and methods

Participants

Recruitment took place between June 2019 to September 2021 and involved inpatients diagnosed with RA who were in the Department of Rheumatology and Immunology wards at the Second Affiliated Hospital of Guizhou University of Traditional Chinese Medicine in southwest China. The inclusion criteria in this study were as follows: 1) compliance with the 2010 American College of Rheumatology/European League Against Rheumatism classification criteria, 2) age $\geq$ 18 years, and 3) clear consciousness and normal cognitive function. The exclusion criteria were: 1) unable to communicate in Chinese well and 2) suffering from other comorbidities.

All participants in this study were residents of Guizhou province. After understanding the procedure and objectives of this study in detail, they formally provided their informed consent in writing, consenting to their participation in this research and granted permission for their information to be published within the confines of this journal. Subsequently, a set of self-reported questionnaires was distributed to all participants, and clinical medical information was gathered from medical records. The total number of recruited participants was 215; however, 19 were ineligible because they did not respond fully to the scales. Therefore, the data of 196 RA patients were included in this study. The sample size fulfilled the recommendation of a minimum of 10 participants per variable to perform path analytic approximations [19].

Methods

Measurement of anxiety and depression. Depression and anxiety were evaluated using the Hospital Anxiety and Depression Scale (HADS). The HADS is the most widely used self-report instrument for detecting anxiety (7 items) and depression (7 items) in medical patients. It is a 4-point scale (0–3), with each subscale's maximum score being 21. Scores of 0–7 indicate "normal," 8–10 indicate a "possible case," and 11–21 suggest a "probable case" of anxiety or

depression [2]. In this study, the Cronbach's α values for the depression and anxiety subscales were 0.73 and 0.81, respectively.

Measurement of resilience. Resilience was measured using the self-reported 10-item Connor–Davidson Resilience Scale (CD-RISC-10). Developed by American psychologists Kathryn M. Connor and Jonathan R. T. Davidson, the CD-RISC-10 originally had a 25-entry, 5-factor structure. Subsequently, a short version with 10 items was developed. Responses to all 10 items range from 0 (never) to 4 (always), with higher scores indicating greater resilience. Research has shown excellent reliability and validity in diverse populations, including community groups, general psychiatric outpatients, and patients with post-traumatic stress disorder and cancer [21–23]; the Cronbach's α value for the CD-RISC-10 was 0.86 in this study.

Measurement of pain. The joint pain intensity caused by RA during the previous week (0 = no pain, 100 = worst possible) was assessed by the visual analog scale (VAS). A VAS score > 40 mm indicates clinically strong pain for RA [8].

Ethical approval. The study was approved by the Ethics Committee of the Second Affiliated Hospital of Guizhou University of Traditional Chinese Medicine (Approval No. KY2019007).

Demographic characteristics and clinical variables

Participants' demographic data (age, gender, marital status, and education level) were recorded on a basic information sheet. Marital status was classified as either married or unmarried, and educational level was categorized as junior middle school and below, senior high school, and junior college or above. Clinical data, including disease duration, TJC28 (tender joint count assessed by 28 joints), SJC28 (swollen joint count assessed by 28 joints), and C-reactive protein (CRP), were collected by trained medical researchers from medical records.

Statistical analyses

Demographic and medical variables are presented as descriptive statistics. Descriptive data analyses were conducted to describe the sample, percentage of categorical data, means, and standard deviations of the continuous data. The Pearson correlation coefficient indicated correlations between the variables. A path analysis model was used to examine the study's hypotheses; path analysis can determine mediation effects and simultaneously estimate the effects among variables [23]. Fig 1 presents a hypothetical path-analytic model with one independent variable, one mediator, and two correlated dependent variables. For our study, pain was an independent variable, resilience was a mediator, and the HAD-Depression and HAD-Anxiety subscales were outcomes. Standardized regression coefficients (β) were calculated based on 5000 bootstrap samples. The indirect effects' parameters were regarded as statistically significant if the 95% confidence interval (CI) did not include 0 [23]. In this study, SPSS version 22.0 and Mplus 7.0 were used. Two-tailed $p < 0.01$ were considered statistically significant. The authors did not have access to information that could identify individual participants during or after data collection. All personal identifiers were removed from the dataset and replaced with unique codes. Additionally, any identifiable information was securely stored and accessible only by the principal investigator, who was responsible for maintaining participant confidentiality. These measures were implemented to maintain the privacy and anonymity of research participants throughout the study.

Results

Sample characteristics

Table 1 shows the demographic and clinical data of patients. The sample comprised 196 patients, of whom 152 (77.6%) were female. Ages ranged from 26 to 82 years (57.35 ± 11.86).

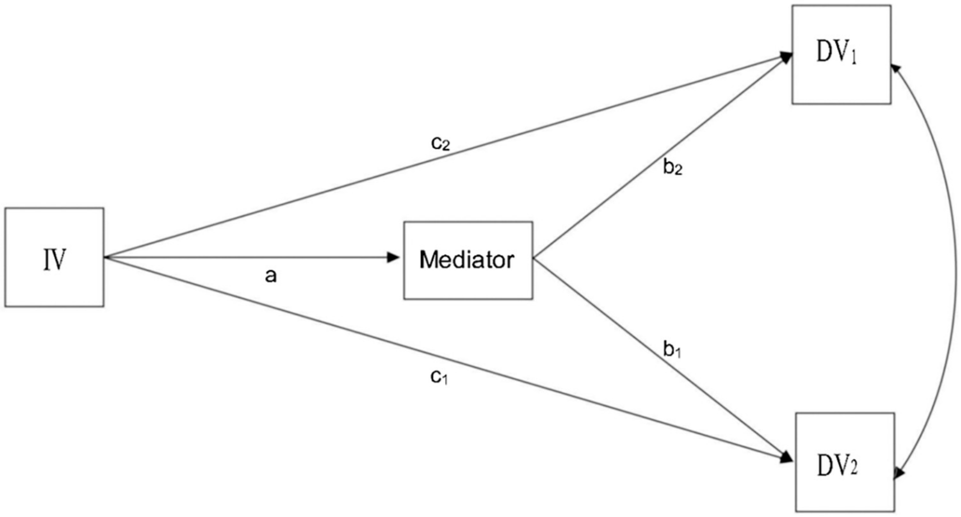

Fig 1. Generic example of a path analysis multiple mediational model. One independent variable (IV), one mediator, and two dependent variables (DV).

https://doi.org/10.1371/journal.pone.0295255.g001

Most participants were married (n = 171, 87%) and had basic education (n = 114, 58.1%). The sample's average disease duration was 10.55 years (SD = 9.86), average VAS-pain score was 36.17 (SD = 6.58), average TJC28 was 7.02 (SD = 3.20), average SJC28 was 1.71 (SD = 1.67),

Table 1. Sociodemographic and clinical characteristics of the sample.

		n = 196	
Sociodemographic variables			
Age (mean, SD; years)		57.35	11.86
Education (n, %)	Junior college or above	25	12.8
	Senior high school	114	58.1
	Junior middle school and below	57	29.1
–Marital status (n, %)	Married	171	87
–Gender (n, %)	Male	44	22.4
	Female	152	77.6
Clinical variables			
Duration of disease (mean, SD; years)		10.55	9.86
VAS-pain (mean, SD)		36.17	6.58
Tender 28-joint counts (mean, SD)		7.02	3.20
Swollen 28-joint counts (mean, SD)		1.71	1.67
C-reactive protein (mean, SD; mg/dl)		13.33	41.07
Depression (mean, SD)		7.48	3.75
	Normal (0–7), (n, %)	93	47.4
	Possibly depressed (8–10), (n, %)	59	30.1
	Probably depressed (≥ 11), (n, %)	44	22.4
Anxiety (mean, SD)		7.62	4.14
	Normal (0–7), (n, %)	102	52
	Possible anxiety (8–10), (n, %)	49	25
	Probable anxiety (≥ 11), (n, %)	45	23
Resilience (CD-RISC) (mean, SD)		25.64	6.15

https://doi.org/10.1371/journal.pone.0295255.t001

Table 2. Pearson correlations among the variables in the multiple mediation analysis.

	Pain	Resilience	Depression	Anxiety
Pain				
Resilience	-0.31**			
Depression	0.59**	-0.54**		
Anxiety	0.26**	-0.38**	0.49**	

**$p < 0.01$.

https://doi.org/10.1371/journal.pone.0295255.t002

and average CRP was 13.33 (*SD* = 41.07). The average HAD-Depression score was 7.48 (*SD* = 3.75), and that of HAD-Anxiety was 7.62 (*SD* = 4.14). Given the cut-off points mentioned above, 44 patients (22.4%) were "probable" depression cases, and 45 (23%) were "probable" anxiety cases. The mean score of CD-RISC (resilience) was 25.64 (*SD* = 6.15).

Bivariate analysis

Table 2 displays the Pearson correlation matrix for the variables employed in the mediation analysis, which includes pain, resilience, depression, and anxiety. All variables exhibited significant correlations with each other at a significance level of $p < 0.01$. The strongest correlation was between pain and depression ($r = 0.59$), followed by depression and resilience ($r = -0.54$).

Mediation model

A path analysis was used to test the mediation model. We further analyzed the specific path coefficients and mediation effects. The partial mediation effect of resilience was significant, with direct effects among the variables demonstrating statistical significance. The path equations representing the direct effects were as follows: Resilience = -0.288 * Pain; Depression = -0.240 * Resilience + 0.268 * Pain; Anxiety = -0.225 * Resilience + 0.097 * Pain. These equations indicated that an increase in resilience was associated with a decrease in pain, depression, and anxiety. This effect of resilience on depression and anxiety was significant (β = -0.39, 95% CI = -0.50 to -0.28, $p < 0.001$ for depression; β = -0.33, 95% CI = -0.47 to -0.19, $p < 0.001$ for anxiety). The indirect effects of resilience on depression and anxiety through pain were also represented by path equations: Depression_Indirect = 0.069 * Pain; Anxiety Indirect = 0.065 * Pain. The indirect effect of resilience on depression through pain was estimated to be 0.069 (95% CI = 0.038 to 0.105, $p < 0.001$), and the indirect effect on anxiety was 0.065 (95% CI = 0.031 to 0.106, $p = 0.001$). The overall indirect effects summed to 0.134, which implies that enhancing resilience can indirectly reduce the impacts of pain on both depression and anxiety. The unstandardized coefficients and standard errors are provided in Table 3, while Fig 2 presents the paths, R^2 values (the proportion of the variance for a dependent variable that is explained by an independent variable or variables), associated standardized coefficients, and 95% CIs (detailed in brackets).

Discussion

In this study, we investigated the underlying mechanisms connecting resilience, pain, and psychological distress (such as depression and anxiety) in hospitalized patients with RA. To our knowledge, this is the first study examining these variables among inpatients with RA through a multiple mediation model. We selected inpatients who needed to be treated in the hospital due to disease severity (mainly judged by the clinical and laboratory variables in Table 1) as

Table 3. Direct and indirect effects in the multiple mediation model.

Direct effects

	Path	β	*SE*	*p* value
Pain → Resilience	a	-0.288	0.057	< .001
Resilience → Depression	b_1	-0.240	0.033	< .001
Pain → Depression	c_1	0.268	0.034	< .001
Resilience → Anxiety	b_2	-0.225	0.046	< .001
Pain → Anxiety	c_2	0.097	0.038	0.012

Indirect effects

	Path	Boots.	*SE*	*p* value	95% CI
Pain → Resilience → Depression	$a \times b_1$	0.069	0.017	< .001	0.038–0.105
Pain → Resilience → Anxiety	$a \times b_2$	0.065	0.020	0.001	0.031–0.106
Total indirect effects		0.134	0.034	<. 001	0.073–0.207

The findings presented in this table are unstandardized, while the standardized results can be found in Fig 2.

https://doi.org/10.1371/journal.pone.0295255.t003

our research sample. Therefore, a study on the mediating role of resilience within this context is of considerable value.

Consistent with previous research [1,2,5], our study found strong associations between RA and depression and anxiety. The findings also highlighted the importance of resilience in patients with RA, aligning with other studies that identified resilience as a key factor in managing pain and psychological distress associated with RA. We utilized a unique methodological approach, employing a single model and path analysis, to examine how resilience mediates the relationship between pain, depression, and anxiety among patients with RA. This approach offers a more comprehensive understanding of the complexities involved in these relationships. Additionally, our study provides valuable insights from a Chinese cultural perspective, which may differ from those found by studies conducted in other cultural contexts. The results of our path analysis model confirmed our hypotheses that pain has a significant direct impact

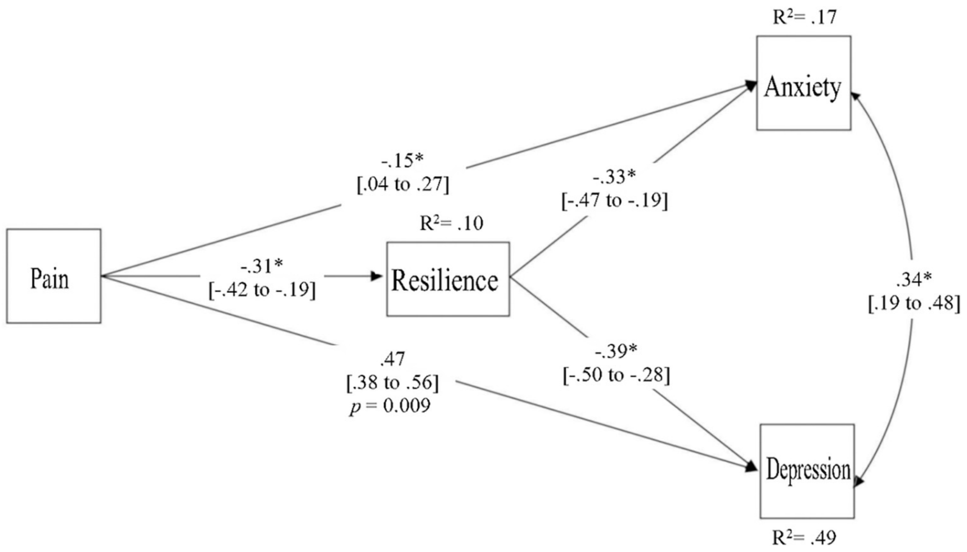

Fig 2. Path analysis multiple mediation mode. $^*p < 0.001$.

https://doi.org/10.1371/journal.pone.0295255.g002

on depression and anxiety, with resilience playing a crucial mediating role. Both direct and indirect effects were found to be significant.

Pain contributed to higher depression and anxiety scores; however, resilience helped mitigate the adverse effects of pain-induced depression and anxiety. These findings align with the proposed roles of resilience counteracting pain [5], depression [24], and anxiety [25]. Chronic pain is recognized as the most distressing symptom in RA [26], with up to 90.4% of patients seeking medical assistance for severe pain [26]. Generally, pain from joint inflammation in RA is viewed as an indicator of elevated disease activity. Some patients experience moderate to severe pain with no signs of inflammation [8]. Prior research has indicated that pain in RA patients is mainly associated with higher anxiety and depression scores [27]. Vergne-Salle et al. [27] found that 38.4% of patients continued to endure moderate to severe pain despite biological treatment, observing a strong correlation between pain and depression and anxiety, which subsequently impacts work, sleep, and mood. Depression and anxiety related to chronic pain are often tied to cognitive and behavioral processes like catastrophizing [1] and passive coping styles [28,29]. As a result, the processes that drive stress responses in RA patients can be subtle [5,30]. However, the way RA patients respond to recurring pain can predict their mental well-being. High resilience in RA patients moderates pain symptoms and the relationship between stress and depressive symptoms. Resilience is considered as a protective factor for RA patients against the negative effects of disease-related stressors on symptom severity, physical function, and psychological quality of life [15]. Moreover, there is ample evidence that resilience prevents or reduces depressive and anxiety symptoms [24,25,31].

Regarding mediating mechanisms, earlier literature has reported that resilience minimizes pain catastrophizing and promotes adjustment to chronic pain, such as active coping [18]. These cognitive properties of resilience may contribute significantly to individual differences in pain perception and responses and reduce the effects of stress on depression and anxiety. Taking into account the adverse impact of chronic pain, the mediating role of resilience demonstrated in this study aligns well with the concept of resilience, enabling individuals to respond positively to disease-related stressors.

Our findings are relevant, as they help elucidate the mechanism that links pain and mental distress. They emphasize the role of resilience as a protective factor for patients with RA suffering from chronic pain. A higher level of resilience can mitigate the negative impacts of pain, consequently leading to lower depression and anxiety levels. The path equations underscore the importance of resilience in mediating the effects of pain on mental health outcomes. This can be explained by the fundamental definition of resilience: the ability to respond positively after exposure to disease-related stressors. Indeed, being resilient does not imply the absence of stress; rather, it means knowing how to deal with suffering and difficulties and finding a way to manage the situation [18]. Thus, resilient patients with RA do not get caught in a vicious cycle of helplessness and hopelessness known to lead to anxiety and depression. The significance of resilience in evaluating and managing the effects of disease and its role as a safeguard against psychological distress in the context of painful conditions should be emphasized. An appropriate management plan should be applied to relieve pain and psychological distress while considering their connections and the critical role of resilience [32].

This study presents certain limitations. First, it is a cross-sectional study, the results of which should be interpreted with caution despite providing valuable insight. In addition, the use of convenience sampling and including participants from only one hospital may restrict the generalization of the study results to the wider RA patient population.

Conclusions

Our study examined the relationship between resilience and pain-induced depression and anxiety in patients with RA using a single model and path analysis. This comprehensive approach allowed us to identify both the direct and indirect effects of resilience on these psychological factors. Conducting the study in China provided valuable insight into the cultural context of RA, contributing to a more holistic understanding of the disease's impact. Clinically, our findings support the implementation of resilience-building interventions for patients with RA to enhance disease adaptation and management strategies. By focusing on resilience, our study addresses an often-overlooked aspect of chronic disease management, filling a critical gap in the existing literature and emphasizing the importance of incorporating resilience into patient care. Additionally, our study calls for further investigation into the influences and mechanisms of resilience in different populations of patients with RA, such as those of different ages, genders, or cultural backgrounds.

Supporting information

S1 Dataset.
(DAT)

Acknowledgments

The authors would like to thank all inpatient participants for their involvement in this study. The authors would also like to extend thanks to their colleagues and friends at Guizhou University of TCM and the Department of Rheumatology and Immunology, Second Affiliated Hospital of Guizhou University of Traditional Chinese Medicine, for their assistance and valuable suggestions.

Author Contributions

Conceptualization: Shuang Xu, Qiongyu Zhang.

Data curation: Shuang Xu, Qiongyu Zhang.

Formal analysis: Shuang Xu.

Investigation: Qiongyu Zhang, Jiayan Zhou.

Resources: Qiongyu Zhang, Jiayan Zhou.

Validation: Shuang Xu.

Writing – original draft: Shuang Xu.

References

1. Edwards RR, Cahalan C, Mensing G, Smith M, Haythornwaite JA. Pain, catastrophizing, and depression in the rheumatic diseases. Rheumatology. 2011; 7(4):216–224. https://doi.org/10.1038/nrrheum.2011.2 PMID: 21283147
2. Covic T, Cumming SR, Pallant JF, Manolis N, Emery P, Conaghan PG, et al. Depression and anxiety in patients with rheumatoid arthritis: Prevalence rates based on a comparison of the Depression, Anxiety and Stress Scale (DASS) and the hospital, Anxiety and Depression Scale (HADS). BMC Psychiatry. 2012; 12:6. https://doi.org/10.1186/1471-244X-12-6 PMID: 22269280
3. Zeng QY, Chen R, Darmawan J, Xiao ZY, Chen SB, Wigley R, et al. Rheumatic diseases in China. Arthritis Res Ther. 2008; 10(1):R17. https://doi.org/10.1186/ar2368 PMID: 18237382

4. Soósová MS, Macejová Ž, Zamboriová M, Dimunová L. Anxiety and depression in Slovak patients with rheumatoid arthritis. J Ment Health. 2017; 26(1):21–27. https://doi.org/10.1080/09638237.2016.1244719 PMID: 27809630
5. Sturgeon JA, Finan PH, Zautra AJ. Affective disturbance in rheumatoid arthritis: psychological and disease-related pathways. Nat Rev Rheumatol. 2016; 12(9):532–542. https://doi.org/10.1038/nrrheum.2016.112 PMID: 27411910
6. Conti Y, Vatine JJ, Levy S, Meltz YL, Hamdan S, Elkana O, et al. Pain catastrophizing mediates the association between mindfulness and psychological distress in chronic pain syndrome. Pain Pract. 2020; 20(7):714–723. https://doi.org/10.1111/papr.12899 PMID: 32285576
7. Gromisch ES, Kerns RD, Beauvais J. Pain-related illness intrusiveness is associated with lower activity engagement among persons with multiple sclerosis. Mult Scler Relat Disord. 2020; 38:101882. https://doi.org/10.1016/j.msard.2019.101882 PMID: 31812040
8. Bilberg A, Bremell T, Bjersing J, Mannerkorpi K. High prevalence of widespread pain in women with early rheumatoid arthritis. Scand J Rheumatol. 2018; 47(6):447–454. https://doi.org/10.1080/03009742.2018.1447683 PMID: 29973088
9. Wolfe F, Michaud K. Predicting depression in rheumatoid arthritis: The signal importance of pain extent and fatigue, and comorbidity. Arthritis Rheumatol. 2009; 61(5):667–673. https://doi.org/10.1002/art.24428 PMID: 19404997
10. Iannuccelli C, Lucchino B, Gioia C, Dolcini G, Favretti M, Franculli D, et al. Mental health and well-being during the COVID-19 pandemic: stress vulnerability, resilience and mood disturbances in fibromyalgia and rheumatoid arthritis. Clin Exp Rheumatol. 2021; 39 Suppl 130(3):153–160. https://doi.org/10.55563/clinexprheumatol/4nb0ku PMID: 34161226
11. Matcham F, Rayner L, Steer S, Hotopf M. The prevalence of depression in rheumatoid arthritis: A systematic review and meta-analysis. Rheumatology. 2013; 52(12):2136–2148. https://doi.org/10.1093/rheumatology/ket169 PMID: 24003249
12. Uguz F, Akman C, Kucuksarac S, Tufekci O. Anti-tumor necrosis factor-alpha therapy is associated with less frequent mood and anxiety disorders in patients with rheumatoid arthritis. Psychiatry Clin Neurosci. 2009; 63(1):50–55. https://doi.org/10.1111/j.1440-1819.2008.01905.x PMID: 19154212
13. Shaul MP. From early twinges to mastery: The process of adjustment in living with rheumatoid arthritis. Arthritis Care Res (Hoboken). 1995: 8(4):290–297. https://doi.org/10.1002/art.1790080414 PMID: 8605269
14. Silverman AM, Molton IR, Alschuler KN, Ehde DM, Jensen MP. Resilience predicts functional outcomes in people aging with disability: A longitudinal investigation. Arch Phys Med Rehabil. 2015; 96(7):1262–1268. https://doi.org/10.1016/j.apmr.2015.02.023 PMID: 25757790
15. Shaw Y, Bradley M, Zhang C, Dominique A, Michaud K, McDonald D, et al. Development of resilience among rheumatoid arthritis patients: A qualitative study. Arthritis Care Res (Hoboken). 2020: 72(9):1257–1265. https://doi.org/10.1002/acr.24024 PMID: 31282121
16. Liu L, Xu X, Xu N, Wang L. Disease activity, resilience and health-related quality of life in Chinese patients with rheumatoid arthritis: a multi-center, cross-sectional study. Health Qual Life Outcomes. 2017; 15:149. https://doi.org/10.1186/s12955-017-0725-6 PMID: 28738816
17. Kim GM, Lim JY, Kim EJ, Park SM. Resilience of patients with chronic diseases: A systematic review. Health Soc Care Community. 2019; 27(4):797–807. https://doi.org/10.1111/hsc.12620 PMID: 30027595
18. Sturgeon JA, Zautra AJ. Resilience: a new paradigm for adaptation to chronic pain. Curr Pain Headache Rep. 2010; 14(2):105–112. https://doi.org/10.1007/s11916-010-0095-9 PMID: 20425199
19. Pérez-Aranda A, García-Campayo J, Gude F, Luciano JV, Soler AF, González-Quintela A, et al. Impact of mindfulness and self-compassion on anxiety and depression: The mediating role of resilience. Int J Clin Health Psychol. 2021; 21(2):100229. https://doi.org/10.1016/j.ijchp.2021.100229 PMID: 33767736
20. Kasser SL, Zia A. Mediating role of resilience on quality of life in individuals with multiple sclerosis: A structural equation modeling approach. Arch Phys Med Rehabil. 2020; 101(7):1152–1161. https://doi.org/10.1016/j.apmr.2020.02.010 PMID: 32169458
21. Campbell-Sills L, Stein MB. Psychometric analysis and refinement of the Connor-Davidson Resilience Scale (CD-RISC): Validation of a 10-item measure of resilience. J Trauma Stress. 2007; 20(6):1019–1028. https://doi.org/10.1002/jts.20271 PMID: 18157881
22. Seiler A, Jenewein J. Resilience in cancer patients. Front Psychiatry. 2019; 10:208. https://doi.org/10.3389/fpsyt.2019.00208 PMID: 31024362
23. Lockhart G, MacKinnon DP, Ohlrich V. Mediation analysis in psychosomatic medicine research. Psychosom Med. 2011; 73(1):29–43. https://doi.org/10.1097/PSY.0b013e318200a54b PMID: 21148809

24. Min JA, Lee CU, Hwang SI, Shin JI, Lee BS, Han SH, et al. The moderation of resilience on the negative effect of pain on depression and post-traumatic growth in individuals with spinal cord injury. Disabil Rehabil. 2014; 36(14):1196–1202. https://doi.org/10.3109/09638288.2013.834985 PMID: 24063294

25. Philippou A, Sehgal P, Ungaro RC, Wang K, Bagiella E, Dubinsky MC, et al. High levels of psychological resilience are associated with decreased anxiety in inflammatory bowel disease. Inflamm Bowel Dis. 2022; 28(6):888–894. https://doi.org/10.1093/ibd/izab200 PMID: 34448855

26. Mathias K, Amarnani A, Pal N, Karri J, Arkfeld D, Hagedorn JM, et al. Chronic pain in patients with rheumatoid arthritis. Curr Pain Headache Rep. 2021; 25(9):59. https://doi.org/10.1007/s11916-021-00973-0 PMID: 34269913

27. Vergne-Salle P, Pouplin S, Trouvin AP, Bera-Louville A, Soubrier M, Richez C, et al. The burden of pain in rheumatoid arthritis: Impact of disease activity and psychological factors. Eur J Pain. 2020; 24 (10):1979–1989. https://doi.org/10.1002/ejp.1651 PMID: 32841455

28. Prell T, Liebermann JD, Mendorf S, Lehmann T, Zipprich HM. Pain coping strategies and their association with quality of life in people with Parkinson's disease: A cross-sectional study. PLoS One. 2021; 16 (11):e0257966. https://doi.org/10.1371/journal.pone.0257966 PMID: 34723975

29. Chiva-Bartoll Ó, Morente-Oria H, González-Fernández FT, Ruiz-Montero P. Anxiety and bodily pain in older women participants in a physical education program. A multiple moderated mediation analysis. Sustainability. 2020; 12:4067.

30. Zautra AJ, Smith BW. Depression and reactivity to stress in older women with rheumatoid arthritis and osteoarthritis. Psychosom Med. 2001; 63(4):687–696. https://doi.org/10.1097/00006842-200107000-00022 PMID: 11485123

31. Nakazawa K, Noda T, Ichikura K, Okamoto T, Takahashi Y, Yamamura T, et al. Resilience and depression/anxiety symptoms in multiple sclerosis and neuromyelitis optica spectrum disorder. Mult Scler Relat Disord. 2018; 25:309–315. https://doi.org/10.1016/j.msard.2018.08.023 PMID: 30176401

32. Santos EF, Duarte CM, Ferreira RO, Pinto AM, Geenen R, da Silva JP. Multifactorial explanatory model of depression in patients with rheumatoid arthritis: a structural equation approach. Clin Exp Rheumatol. 2019; 37(4):641–648. PMID: 30418126
